# Intentional Degradation for Stabilizing Residual Learning in Coarse-to-Fine Medical Image Refinement

**DOI:** 10.3390/jimaging12090418

**Published:** 2026-09-05

**Authors:** Myongjin Kim, Shin Ae Lee, Hasung Kim, Seontai Park, Jongsoo Park, Jaechul Yoon, Yong Suk Cho, Jun Hur, Dohern Kym

**Affiliations:** 1 Department of Burn Surgery, Hangang Sacred Heart Hospital, Hallym University College of Medicine, Seoul 07247, Republic of Korea; pacta300@hallym.or.kr (M.K.);; 2 Department of Surgery, Burn Treatment Center, Pohang St. Mary’s Hospital, Pohang 37661, Republic of Korea

**Keywords:** intentional degradation, residual learning, medical image refinement, coarse-to-fine generation, distribution alignment, image synthesis stability

## Abstract

**Background:** Two-stage coarse-to-fine architectures are commonly used in medical image synthesis and refinement. However, when the distributional gap between coarse inputs and high-resolution targets is large, the second-stage generator may produce unstable or weakly conditioned refinements rather than operating as a true residual corrector. **Methods:** We propose intentional degradation, a target-side distribution-alignment strategy that deliberately degrades high-resolution targets toward the coarse prediction space before residual learning. The degradation profile is guided by measured differences in contrast, saturation, and edge energy. We further evaluate refinement quality using two residual-space metrics: Var(Δ), the variance of the predicted residual (target minus input), and Δ-SSIM, the structural similarity between the predicted and true residual maps; both are designed to reveal refinement instability that conventional image-level metrics (e.g., SSIM computed on the full image) may not capture. We evaluated the approach in three medical imaging domains: wound-healing photography, retinal fundus imaging, and dermoscopy. **Results:** Across all three domains, intentional degradation consistently improved Δ-SSIM and normalized residual variance recovery relative to the baseline, and predicted residuals showed substantially stronger directional correspondence with the true residual (assessed via sign-agreement and cosine-similarity metrics) than the baseline, which converged toward directionally random predictions. These findings suggest that residual-space and direction-sensitive metrics can reveal refinement instability that may not be captured by conventional image-level metrics alone. **Conclusions:** Intentional degradation provides a simple, architecture-agnostic-by-design strategy for stabilizing residual learning in coarse-to-fine medical image refinement, validated within a single ConvLSTM-based refinement framework; extension to other architectures remains for future validation. Rather than introducing a new network architecture, the method modifies the target-side training distribution to reduce the mismatch between coarse inputs and high-resolution targets.

## 1. Introduction

Two-stage coarse-to-fine architectures have become a dominant paradigm in medical image generation [1,2,3,4]. Many generative pipelines adopt a coarse-to-fine design, in which an initial model produces a low-fidelity coarse representation and a subsequent generator enhances realism by adding high-frequency detail. Although this approach is conceptually straightforward, such systems frequently suffer from a fundamental coarse–fine distribution mismatch.

During training, the second-stage generator is supervised to map a statistically weak coarse input to a fully high-quality target image. When the gap between these distributions is large—e.g., differences in contrast, texture complexity, dynamic range, or edge sharpness—the generator may minimize the training objective more effectively by ignoring the coarse input and synthesizing a high-fidelity image directly from its internal priors. This failure mode is particularly problematic in conditional coarse-to-fine pipelines, where the second-stage generator is expected not merely to improve visual realism, but to remain structurally conditioned on the coarse prediction produced by the first-stage model.

This behavior leads to generator collapse: instead of performing residual correction or detail enhancement, the generator functions as an almost unconditional image synthesizer, discarding the structural information carried by the coarse stage. As a result, training becomes unstable, consistency with the low-resolution (LR) or coarse input is lost, and the intended benefits of the coarse-to-fine design fail to materialize.

**Figure 1:**
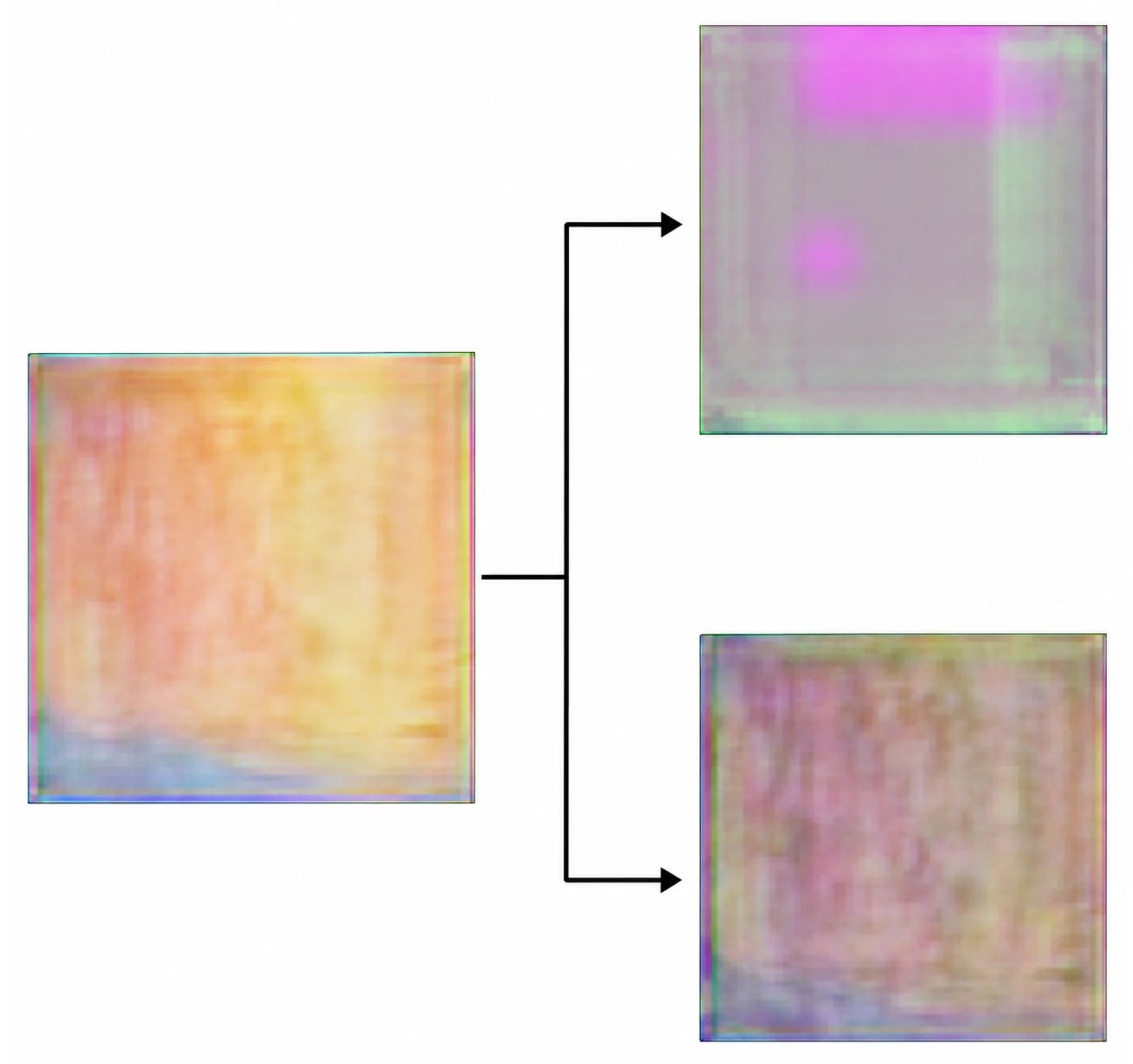
Ablation study of intentional degradation using genuine Creator outputs as coarse inputs. Both outputs originate from the same coarse prediction produced by the first-stage generative model (**left**). (**Top**) Without intentional degradation, the second-stage generator bypasses the coarse input and produces an output that diverges structurally and chromatically—a visual manifestation of generator collapse. (**Bottom**) With intentional degradation applied during training, the generator remains conditioned on the coarse structure and produces a stable, structurally consistent refinement. This result motivated the development and systematic cross-domain evaluation of the intentional degradation training strategy described in this study.

To address the instability of conditional coarse-to-fine generators, we propose intentional degradation as a training strategy that aligns the target distribution with the coarse prediction space. This failure mode was directly observed in a clinical wound-healing forecasting pipeline using genuine Creator outputs, as illustrated in Figure 1, motivating the systematic cross-domain evaluation described in this study. Because this pipeline is intended to support clinical decision-making regarding wound care, generator collapse is not merely a technical artifact: an unstable or unconditioned refinement can propagate a misleading healing trajectory forecast, directly affecting the clinical utility of the system.

Despite being widely observed in practice, this failure mode has not been explicitly formalized or quantitatively characterized in prior work. Existing stabilization strategies, such as perceptual losses, feature normalization, or adversarial regularization [5], may improve visual quality but do not directly address the underlying mismatch between the coarse input distribution and the target distribution.

To address this gap, we introduce Intentional Degradation, a simple yet principled method that intentionally degrades high-resolution target images during training so that their statistics match those of the coarse prediction space. By aligning the target distribution with the coarse input distribution, the second-stage generator is encouraged to operate as a true residual corrector, learning clinically meaningful deltas instead of re-synthesizing the entire image.

Based on this observed failure mode, we formulate generator collapse as a distributional mismatch between the coarse prediction space and the high-resolution target space, and evaluate the proposed strategy across three medical imaging domains: wound-healing photography, retinal fundus imaging (DRIVE), and dermoscopy (ISIC 2018).

Beyond the originating wound-healing application, we selected retinal fundus imaging and dermoscopy as evaluation domains because both are central to high-volume clinical screening pipelines—diabetic retinopathy detection from fundus photography and melanoma triage from dermoscopic imaging—where a coarse-to-fine refinement stage prone to collapse (either through re-synthesis or hallucinated content) carries direct diagnostic risk rather than serving only as a generalization benchmark.

In summary, the novelty of this work is threefold: (1) we identify and formalize a target-side distributional mismatch as a distinct cause of generator collapse in coarse-to-fine medical image refinement, complementary to prior architecture-based solutions; (2) we introduce intentional degradation, a lightweight, empirically calibrated target-side intervention that requires no architectural modification; and (3) we introduce direction-sensitive residual-space metrics (sign-agreement and cosine-similarity between predicted and true residuals) that reveal a collapse mechanism—direction-agnostic residual prediction—invisible to conventional image-quality or magnitude-based residual metrics alone.

We situate this contribution relative to recent target-side perturbation strategies explored in diffusion modeling and physics-informed image restoration in Section 4.2.

## 2. Methods

### 2.1. Overview and Problem Formulation

**Figure 2:**
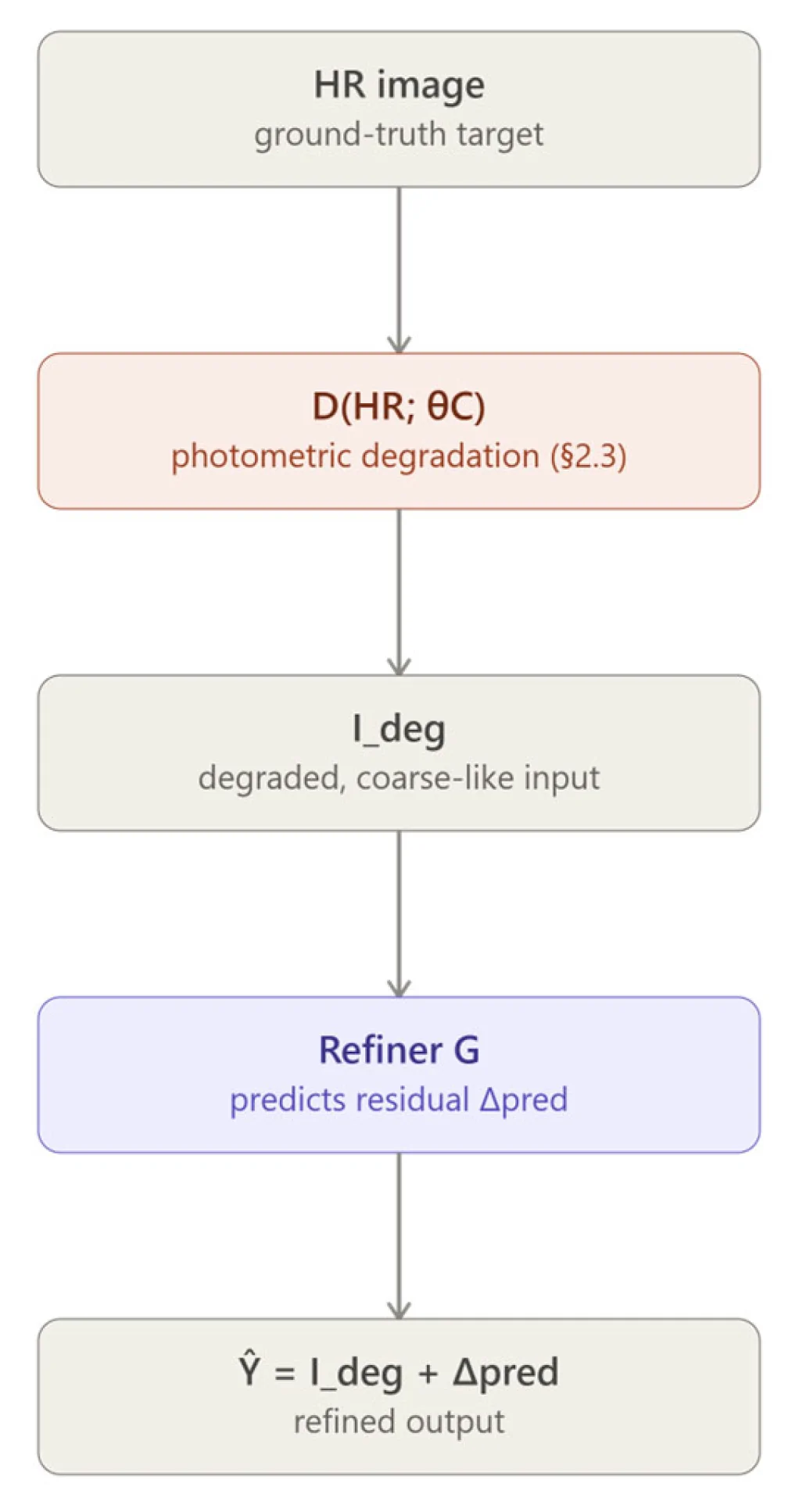
Overview of the intentional degradation training pipeline. A high-resolution (HR) target image is deliberately degraded using the operator D(HR; θC) (Section 2.3), whose parameters are calibrated from HR-coarse statistical profiling (Section 2.2), producing a degraded, coarse-like input I_deg. The refiner network G is trained to predict the residual Δpred required to recover the HR target from I_deg, and the final refined output Ŷ is obtained by adding this predicted residual back to I_deg. Training minimizes a composite loss between Δpred and the ground-truth residual Δ* = HR − I_deg (Section 2.4); evaluation additionally examines the spatial alignment between Δpred and Δ* (Section 2.5).

The remainder of this section is organized as follows. Section 2.2 describes how the statistical gap between coarse predictions and high-resolution targets is measured. Section 2.3 introduces the degradation operator that uses this profile to construct training targets, and formalizes the resulting residual learning [6] objective. Section 2.4 specifies the training loss. Section 2.5 defines the residual-space and spatial alignment metrics used for evaluation. Figure 2 summarizes this pipeline.

This study was motivated by a real coarse-to-fine wound-healing forecasting pipeline in which a ConvLSTM-based first-stage generative model—hereafter referred to as the Creator—produced coarse future wound images. These Creator outputs were not generated by degrading high-resolution images; rather, they were independent coarse predictions produced from preceding wound image sequences. Although they preserved broad wound structure, they were statistically weaker than HR clinical images, with reduced contrast, attenuated saturation, and weakened edge responses.

To isolate and evaluate the collapse mechanism in a controlled setting, we constructed coarse inputs by intentionally degrading HR images so that the resulting images statistically resembled the observed coarse prediction space. Let D denote the intentional degradation operator, and let θ_C represent the degradation parameters selected based on the empirical characteristics of coarse Creator outputs, including contrast, saturation, intensity distribution, and edge-energy attenuation.

**Figure 3:**
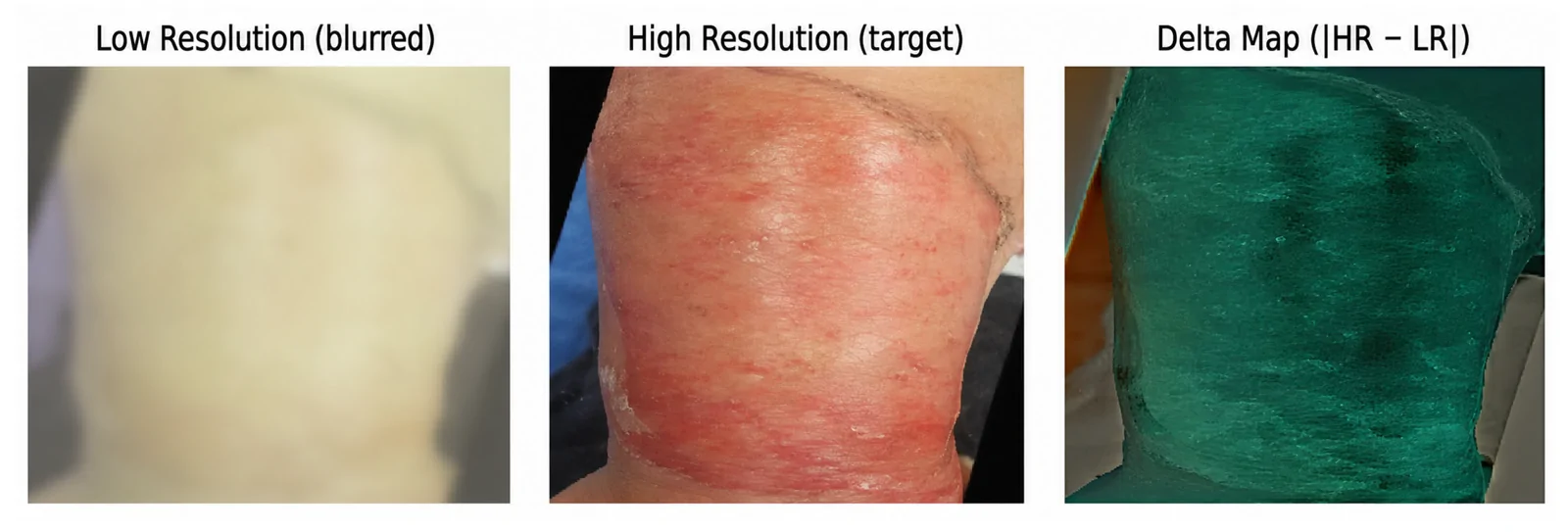
Illustration of the intentional degradation strategy. (**Left**) Low-resolution input generated by intentionally degrading the high-resolution target, simulating severe information loss such as blurring. (**Middle**) High-resolution target image. (**Right**) Delta map (|HR − LR|), visualizing the high-frequency details and textural information lost during the degradation process—the residual signal that the generator is trained to recover.

For each high-resolution target image HR, we generated a degraded coarse surrogate (Figure 3):

*I_deg* = *D*(*HR*; *θ_C*)

The ground-truth residual was then defined as:

Δ* = *HR* − *I_deg*

The refinement generator G received the degraded coarse surrogate and predicted the missing residual component:

Δ_*pred* = *G*(*I_deg*)

The refined output was reconstructed by residual addition:

*Ŷ* = *I_deg* + Δ_*pred*

The generator was optimized in residual space:

*L_res* = *L*(Δ_*pred*, Δ*)

with additional perceptual, edge-aware, and sharpening terms described below.

Importantly, D(HR; θ_C) was used as a controlled training and evaluation construct, not as an inference-time replacement for a real Creator output. In a deployed coarse-to-fine forecasting pipeline, the coarse input would be produced directly by the first-stage Creator:

*C* = *Creator*(*x*)

and the refined output would be obtained by applying the residual correction to that coarse prediction:

*Ŷ* = *C* + *G*(*C*)

The present study focuses on whether aligning the target-side degradation distribution with the coarse prediction space can prevent second-stage generator collapse under controlled residual-refinement conditions.

### 2.2. Creator-Informed Statistical Profiling of the Coarse Prediction Space

The effectiveness of intentional degradation relies on accurately characterizing the distributional gap between high-resolution ground-truth images and their corresponding coarse predictions. Rather than assuming a generic low-quality distribution, we explicitly measure the gap statistics of the originating clinical domain to construct an empirically grounded degradation profile. The degradation operator was not chosen as a generic blur or arbitrary low-quality transformation. Instead, it was designed to approximate the statistical profile of the coarse Creator outputs that motivated this study.

For the wound-healing domain, the degradation profile was informed by the empirical characteristics of observed Creator outputs. For DRIVE and ISIC 2018, where no task-specific Creator was trained, degraded coarse surrogates were generated using the same controlled degradation framework to evaluate transferability across external modalities.

Specifically, given pairs of high-resolution ground-truth images and their corresponding coarse predictions, we characterize the distributional gap by computing statistics across both distributions. The measured discrepancy guides the design of the intentional degradation operator:

- Intensity statistics: per-channel RGB intensity distributions and global luminance histograms
- Contrast and saturation: spatial contrast and color saturation distributions
- Edge characteristics: edge-energy spectra computed using Sobel and Gabor filters

These measurements reveal that coarse predictions are systematically lower in contrast and saturation, exhibit attenuated high-frequency components, and contain weakened edge responses compared to ground-truth images. We treat the measured distributional gap as a design specification for the intentional degradation operator. By compressing the HR target distribution toward the coarse prediction space by an amount proportional to the observed gap, we ensure that the residual learning problem presented to the generator is well-conditioned and statistically aligned with the coarse input.

Table 1 summarizes the measured gap statistics across the three evaluated domains.

### 2.3. Intentional Degradation and Residual Learning Pipeline

Using the statistical profile derived in Section 2.2 for the Wound domain, we construct an intentional degradation pipeline that transforms each ground-truth image into a degraded counterpart whose distribution approximates that of the coarse predictions. Concretely, the degradation operator is defined as the following composition:

D(HR; θ_C) = S(B(HR; σ); c, s, δ_h, γ) (1)

where B(·; σ) applies Gaussian blur with kernel parameter σ, and S(·) sequentially applies:

I_1_ = adjust_contrast(B(HR; σ), c), c = 0.7

I_2_ = adjust_saturation(I_1_, s), s = 0.6

I_3_ = adjust_hue(I_2_, δ_h), δ_h~U(−0.10, 0.10)

I_4_ = I_3_^γ, γ = 1.05

I_deg = (1 − β)·I_4_ + β·avgpool(I_4_), β = 0.3

The contrast and saturation coefficients (c, s) were calibrated once on the Wound domain by matching the measured HR–coarse gap statistics in Table 1. The hue perturbation δ_h is sampled per-image from a fixed uniform range rather than a single constant value; this design choice was adopted after we found in preliminary experiments that a fixed hue-shift constant allowed the network to partially memorize a single fixed color-correction direction rather than learning a genuine target-adaptive residual (see Section 4.4). With the exception of this per-image hue sampling, all degradation parameters (σ, c, s, γ, β) are fixed constants determined prior to training and remain unchanged throughout the training process; none are adapted or updated based on model performance or intermediate results during training.

For DRIVE and ISIC 2018, the same parameter values (c = 0.7, s = 0.6, δ_h~U(−0.10, 0.10), γ = 1.05, β = 0.3) were applied without domain-specific re-calibration, despite substantial differences in baseline image statistics across domains (Table 1; e.g., HR contrast ranges from 0.295 for ISIC to 0.888 for DRIVE). This reuse was not originally designed as a formal cross-domain generalization test; we report it here as an informative post-hoc observation that a single degradation profile, calibrated once on one imaging domain, remained effective when applied unmodified to substantially different domains (Section 3.3). A systematic ablation isolating the contribution of each individual degradation component (contrast-only, saturation-only, blur-only, full combination) was not performed in this study and is identified as an important direction for future work (Section 4.4).

Given this degraded target, the generator is trained to predict the residual Δ = HR − I_deg, and the final refined output is obtained as Ŷ = I_deg + Δ_pred. This formulation fundamentally alters the generator’s optimization landscape. Instead of learning to synthesize an entire image from a mismatched coarse input, the generator is constrained to recover only the missing high-frequency details encoded in the residual.

### 2.4. Training Objectives

The generator is trained to recover the residual signal between the intentionally degraded target and the original high-resolution image. The overall training objective is:

L_total = λ1·L1(Δ_pred, Δ*) + λ2·L2(Δ_pred, Δ*) − λ3·std(Δ_pred)

+ λ4·L_ch-std(Δ_pred) + λ5·L_percep(HR, Ŷ)

where Δ_pred = G(I_deg) is the predicted residual and Δ* = HR − I_deg is the ground-truth residual. The L1 and L2 terms encourage accurate recovery of the residual signal. The negative standard deviation term (weighted by λ3) discourages the generator from collapsing toward a near-constant, trivial residual. The channel-wise standard deviation term (λ4) further regularizes the per-channel spread of the predicted residual. The perceptual term (λ5), computed between the refined output Ŷ = clip(I_deg + Δ_pred, 0, 1) and the original HR image using a VGG-based feature representation, encourages perceptual realism in the final refined output. In practice, we found this perceptual term to dominate the total gradient magnitude by several orders of magnitude across most of training (Section 3.4); we verified that the collapse-prevention behavior of the method is not an artifact of this imbalance (Section 3.4).

The weights (λ1 = 1.0, λ2 = 0.5, λ3 = 0.05, λ4 = 0.3, λ5 = 0.3) were selected through limited manual tuning on the Wound domain prior to the full 3-seed runs, guided by two criteria: (i) each term should produce a non-degenerate gradient signal (verified by monitoring per-term loss magnitudes during early training, as in Section 3.4), and (ii) the combination should yield stable convergence in preliminary single-seed trials without oscillation or divergence in the total loss. The same weights were subsequently reused across all three domains without per-domain re-tuning, consistent with the fixed-parameter design described for the degradation operator (Section 2.3), as summarized in Algorithm 1. We did not conduct a systematic hyperparameter sweep or sensitivity analysis over these weights, which we identify as a direction for future work (Section 4.4).
**Algorithm 1:** Intentional Degradation TrainingInput: HR training set {HR_i}, degradation parameters θ_C = (σ, c, s, δ_h, γ, β) Output: Trained refiner G 
1:for each HR_i in training set do2:  I_deg,i ← D(HR_i; θ_C)         //degrade target (Equation (1), Section 2.3)3:  Δ*_i ← HR_i − I_deg,i            //ground-truth residual4:end for5:for epoch = 1 to E do6:  for each mini-batch (I_deg, Δ*) do7:       Δ_pred ← G(I_deg)             //predicted residual8:       Ŷ ← clip(I_deg + Δ_pred, 0, 1)       //refined output9:       L ← λ1·L1(Δ_pred, Δ*) + λ2·L2(Δ_pred, Δ*)       − λ3·std(Δ_pred) + λ4·L_ch-std(Δ_pred)       + λ5·L_percep(HR, Ŷ)           //total loss (§2.4)10:     Update G via ∇L11:   end for12:   if epoch mod 5 == 0 then13:     compute sign_match, cosine_sim on held-out split (§2.5)14:   end if15:end for16:return G

### 2.5. Residual-Space Evaluation Metrics

Because generator collapse in coarse-to-fine refinement may not be adequately captured by conventional pixel-level metrics alone, we evaluated model behavior using residual-space metrics. In this study, the ground-truth residual was defined as the difference between the high-resolution target and the degraded coarse surrogate:

Δ* = *HR* − *I_deg*

The predicted residual was defined as the output of the refinement generator:

Δ_*pred* = *G*(*I_deg*)

The final refined image was reconstructed as:

*Ŷ* = *I_deg* + Δ_*pred*

We used the following metrics to characterize residual learning stability and collapse suppression.

SSIM was calculated between the reconstructed image Ŷ and the high-resolution target HR to assess overall structural similarity at the image level. However, SSIM alone may be misleading in collapse-prone refinement settings, because a generator that re-synthesizes an HR-like image may obtain a high SSIM while ignoring the coarse input.

Δ-SSIM was therefore used to measure structural similarity in residual space:

Δ-*SSIM* = *SSIM*(Δ_*pred*, Δ*)

Higher Δ-SSIM indicates that the predicted residual preserves the structure of the true missing information rather than producing arbitrary or hallucinated corrections. Values near zero suggest poor residual alignment, whereas positive values indicate increasingly faithful residual recovery.

Var(Δ) was used to quantify the variance of the predicted residual map:

*Var*(Δ) = *Var*(Δ_*pred*)

Excessively unstable residual predictions indicate that the generator is not operating as a constrained residual corrector. A progressive reduction in Var(Δ), when accompanied by improved Δ-SSIM, suggests stabilization of residual learning. However, Var(Δ) should not be interpreted in isolation, because a trivially near-zero residual may also have low variance.

WΔ denotes the Wasserstein distance between the predicted residual distribution and the ground-truth residual distribution:

WΔ = W(Δ_pred, Δ*)

Lower WΔ indicates closer distributional alignment between the predicted and true residuals. In the context of intentional degradation, decreasing WΔ suggests that the generator is learning the residual distribution induced by the degraded coarse surrogate rather than drifting toward unconditional image synthesis.

To enable fair comparison of residual variance across conditions with intentionally different target scales, we additionally report a normalized residual variance:

Var(Δ)_norm = Var(Δ_pred)/Var(Δ*)

where a value near 1.0 indicates that the predicted residual fully recovers the variance of the target residual, and departures from 1.0 indicate over- or under-dispersed residual predictions. This normalization corrects a confound present in raw Var(Δ) comparisons, whose scale differs systematically between conditions due to the degradation-induced gap in target variance.

We further assessed the structural (as opposed to purely statistical) correspondence between predicted and ground-truth residuals using two sign/direction-based metrics. Sign match is the fraction of corresponding pixels for which the predicted and ground-truth residuals share the same sign:

sign_match = mean(sign(Δ_pred) == sign(Δ*))

Cosine similarity is computed between the flattened predicted and ground-truth residual vectors:

cosine_sim = (Δ_pred · Δ*)/(||Δ_pred|| ||Δ*||)

For both metrics, values near 0.5 (sign_match) and 0 (cosine_sim) indicate that the predicted residual is statistically indistinguishable from a random direction relative to the true residual, whereas values approaching 1.0 indicate genuine structural correspondence.

Together, Δ-SSIM, Var(Δ)_norm, WΔ, sign_match, and cosine_sim provide complementary information: Δ-SSIM and sign_match/cosine_sim evaluate residual structural fidelity and directional correspondence, Var(Δ)_norm evaluates residual variance recovery relative to the target, and WΔ evaluates residual distributional alignment.

To assess statistical significance despite the limited number of independent runs (n = 3 seeds per domain), we conducted paired *t*-tests comparing With ID and Without ID conditions at matched seeds, both within each domain and pooled across all three domains (n = 9). We additionally report the non-parametric Wilcoxon signed-rank test, which does not assume normality, as a robustness check given the small per-domain sample size.

## 3. Results

### 3.1. Experimental Setup

We evaluated the proposed strategy in one originating in-domain setting and two external cross-modality settings. The wound-healing photography dataset represents the clinical domain in which coarse-to-fine generator collapse was initially observed. For controlled evaluation, high-resolution wound images were intentionally degraded to generate coarse surrogate inputs, allowing residual refinement behavior to be quantified independently of the original Creator architecture.

The evaluated datasets were as follows:

- Wound-healing dataset (N = 451; natural-light wound photography), representing the originating in-domain evaluation setting
- DRIVE retinal fundus dataset (N = 40; fundus photography; training and test sets combined), included as a small-scale cross-modality signal verification setting rather than a large-scale generalization benchmark
- ISIC 2018 dermoscopy dataset (N = 500; randomly sampled from the Task 3 training set), representing an external cross-modality evaluation setting

These domains were selected to represent substantially different image characteristics, including imaging modality, color distribution, edge structure, and clinical context. The wound-healing dataset was included as the originating clinical domain, whereas DRIVE and ISIC 2018 were used to examine whether the proposed strategy was not specific to wound photography. For the wound-healing domain, degradation parameters were informed by the empirical characteristics of the observed Creator output distribution. For DRIVE and ISIC 2018, the same degradation framework was applied in a controlled surrogate-refinement setting to evaluate transferability across external modalities.

For held-out validation, images were split at the level of source identity rather than individual (possibly augmented) samples: for the Wound domain, a group-aware split (GroupShuffleSplit) was used to prevent augmented copies of the same source image from appearing in both training and validation sets, following the discovery of such leakage in an earlier data-loading configuration; for DRIVE, all 40 available images (training and test folders combined) were used, after correcting an earlier loader configuration that inadvertently used only half this set.

Implementation details. All models were trained using the Adam optimizer with an initial learning rate of 1 × 10^−5^, reduced by a factor of 0.5 upon loss plateau (patience of 5 epochs, minimum learning rate 1 × 10^−7^). Batch size was fixed at 16 across all domains. Each configuration (With ID/Without ID) was trained for 100 epochs and repeated across 3 independent random seeds (42, 43, 44) per domain, yielding 18 total training runs. Training was performed on a single NVIDIA L4 GPU (Google Colab). Approximate wall-clock training time per run was 2 h for the Wound and ISIC domains and 20 min for the DRIVE domain, reflecting differences in dataset size. No separate held-out validation set was used during refinement-generator training; all available images within each domain were used for training, and model behavior was monitored via the residual-space metrics described in Section 2.5, computed on a fixed evaluation batch at each epoch. This design choice and its implications are discussed further in Section 4.4 (Limitations).

### 3.2. Prevention of Generator Collapse

Figure 1 illustrates the collapse mechanism using genuine Creator outputs as coarse inputs—independent coarse predictions produced by the first-stage generative model from wound image sequences, not controlled surrogates. Without intentional degradation, the generator ignores the Creator output and reproduces the HR target directly; with intentional degradation, the generator remains conditioned on the coarse structure and produces a stable, structurally faithful refinement. This result confirms that the collapse phenomenon occurs under real coarse-to-fine inference conditions and motivated the systematic cross-domain quantitative evaluation described below.

**Figure 4:**
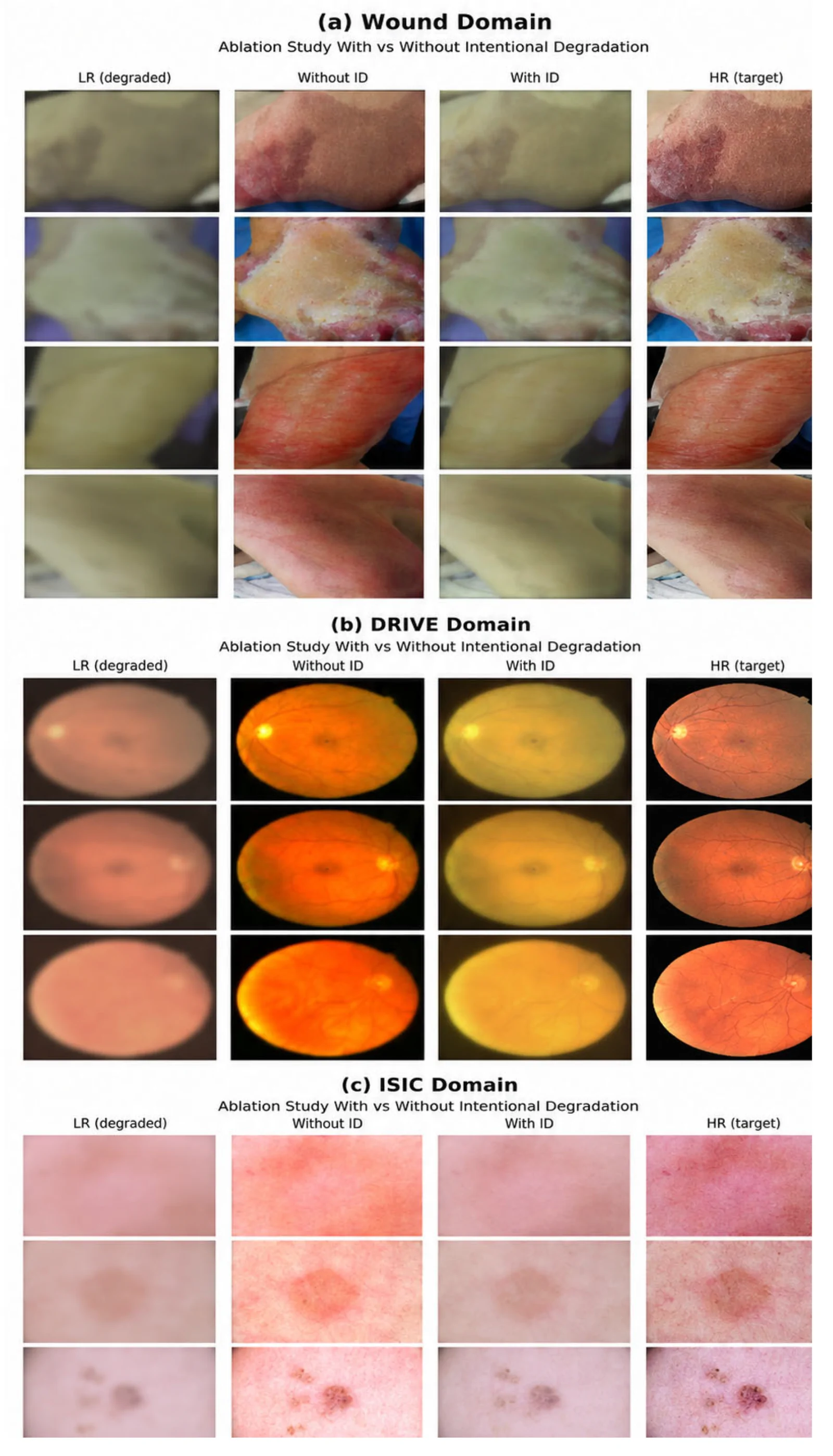
Cross-domain visualization: With vs. Without Intentional Degradation. (**a**) Wound ((**left**) to (**right**): left thigh, lateral aspect; left hand dorsum; left thigh, anterior aspect; left leg); (**b**) DRIVE; (**c**) ISIC. For each domain, the same degraded input (column 1) is processed under two conditions. Without ID (column 2): the generator produces outputs structurally inconsistent with the LR input—in the Wound domain, outputs closely resemble HR ground truth (memorization collapse); in the DRIVE domain, outputs exhibit progressive color distortion (degradation collapse); in the ISIC domain, outputs show color-prior hallucination with over-saturated tones. With ID (column 3): the generator preserves structural fidelity to the LR input while recovering meaningful details across all three domains. HR ground truth is shown for reference (column 4).

Figure 4 extends this comparison across all three domains. Generator collapse manifests in domain-specific ways:

- Wound domain (N = 451): collapse takes the form of HR memorization, producing high SSIM scores that reflect re-synthesis rather than refinement.
- DRIVE domain (N = 40): limited data prevents memorization; collapse manifests as progressive performance degradation over 100 epochs [see Section 3.2 for updated 3-seed statistics].
- ISIC domain (N = 500): collapse produces color-prior hallucination—outputs with distorted saturation unrelated to the LR input structure.

Without ID, the mechanism underlying this collapse is clarified by the spatial alignment analysis (Section 3.3): across all three domains, the predicted residual’s direction becomes statistically indistinguishable from chance (sign_match ≈ 0.5, cosine_sim ≈ 0), despite non-trivial residual magnitude in most domains (Table 2). This indicates that the generator settles into a direction-agnostic solution that reduces training loss without learning content-adaptive structure, rather than uniformly reproducing the HR target (Wound), destabilizing over time (ISIC), or failing solely due to limited data (DRIVE)—domain-specific surface behaviors (Figure 4) share this common underlying failure mode.

**Table 2:** With vs. Without Intentional Degradation—cross-domain residual-space comparison on training data (3-seed mean ± SD, hue-randomized degradation, 100 epochs per run).

Domain	Condition	Δ-SSIM (Mean ± SD, n = 3)	Var(Δ)_Norm (Mean ± SD, n = 3)	WΔ
Wound	With ID (proposed)	0.491 ± 0.011	0.844 ± 0.038	0.0163 ± 0.0009
Wound	Without ID	0.444 ± 0.016	0.549 ± 0.024	0.0067 ± 0.0007
ISIC	With ID (proposed)	0.464 ± 0.002	0.951 ± 0.030	0.0246 ± 0.0054
ISIC	Without ID	0.406 ± 0.008	1.443 ± 0.816 *	0.0090 ± 0.0026
DRIVE	With ID (proposed)	0.286 ± 0.056	N/A **	0.0583 ± 0.0077
DRIVE	Without ID	0.097 ± 0.065	N/A **	0.0592 ± 0.0371

Var(Δ)_norm = Var(Δ_pred)/Var(Δ*), the predicted residual variance normalized by the target residual variance (1.0 = perfect variance recovery). This normalization corrects for the confound in raw Var(Δ) drop, whose scale differs systematically between conditions due to the target-variance gap intentionally introduced by degradation. * ISIC Without ID shows elevated variance across seeds (one seed contributing a markedly higher ratio); reported as a point estimate with the caveat that seed-to-seed variability is substantial. ** DRIVE Var(Δ)_norm omitted: per-seed target variance Var(Δ*) was estimated from the full dataset rather than each seed’s own train/validation partition, and the resulting normalized ratios showed standard deviations exceeding their means (With ID: 0.503 ± 0.445; Without ID: 1.653 ± 1.409)—a sign the estimate is unreliable given DRIVE’s small sample size (N = 40). We therefore rely on the held-out validation and spatial alignment metrics in Table 3, which were computed consistently within each seed’s own split, as the primary evidence for DRIVE.

**Table 3:** Held-out validation and structural (spatial) alignment of predicted residuals, With vs. Without Intentional Degradation (3-seed mean ± SD, 100 epochs per run). Held-out split constructed at the level of source images (group-aware for Wound, to prevent augmentation-induced leakage); DRIVE evaluated on the corrected full dataset (N = 40).

Domain	Condition	Held-Out Val Δ-SSIM (Mean ± SD, n = 3)	Sign_Match (Mean ± SD, n = 3)	Cosine_Sim (Mean ± SD, n = 3)
Wound	With ID (proposed)	0.291 ± 0.009	0.787 ± 0.008	0.667 ± 0.017
Wound	Without ID	0.191 ± 0.002	0.519 ± 0.001	0.055 ± 0.006
DRIVE	With ID (proposed)	0.301 ± 0.054	0.862 ± 0.033	0.709 ± 0.022
DRIVE	Without ID	0.106 ± 0.090	0.489 ± 0.016	0.021 ± 0.029
ISIC	With ID (proposed)	0.426 ± 0.031	0.827 ± 0.011	0.769 ± 0.037
ISIC	Without ID	0.372 ± 0.015	0.523 ± 0.008	0.032 ± 0.008

Note: sign_match is the fraction of pixels where predicted and ground-truth residuals share the same sign (0.5 = chance level); cosine_sim is the cosine similarity between flattened predicted and ground-truth residual vectors (0 = no linear structural correspondence). Both metrics computed at the final training epoch (epoch 100) on held-out data across three domains × three seeds (9 runs total per condition).

Despite these domain-specific collapse mechanisms, intentional degradation consistently mitigates collapse-like instability across all three domains.

Table 4 summarizes early-epoch residual-space metrics under intentional degradation for a representative Wound-domain run, including the spatial alignment metrics introduced in Section 3.3.

Table 2 presents the cross-domain With vs. Without ID comparison, reported as 3-seed mean ± SD.

### 3.3. Spatial Alignment of Predicted Residuals

While Δ-SSIM and Var(Δ)_norm characterize the overall fidelity and scale of predicted residuals, they do not directly test whether the predicted residual is spatially coherent with the true residual beyond what magnitude or global structure alone would suggest. We therefore examined two direction-sensitive metrics—sign_match and cosine_sim (defined in Section 2.5)—computed on held-out data at the final training epoch, across three seeds per domain (Table 3).

Across all three domains, Without ID consistently converged toward sign_match values statistically indistinguishable from chance (Wound: 0.519 ± 0.001; DRIVE: 0.489 ± 0.016; ISIC: 0.523 ± 0.008) and cosine_sim values near zero (Wound: 0.055 ± 0.006; DRIVE: 0.021 ± 0.029; ISIC: 0.032 ± 0.008), indicating that the predicted residual’s direction was effectively uncorrelated with the true residual despite non-trivial residual magnitude in most domains. With ID, both metrics were substantially and consistently elevated (sign_match: 0.787–0.862; cosine_sim: 0.667–0.769), indicating genuine structural correspondence between predicted and true residuals. This pattern was reproduced in 9 of 9 seed-domain combinations (three domains × three seeds each), suggesting that it reflects a general property of the training dynamics rather than a domain- or seed-specific artifact.

This result clarifies a mechanism that Δ-SSIM and Var(Δ)_norm alone cannot distinguish: rather than failing to produce a residual of reasonable magnitude, the baseline (Without ID) model produces a residual whose direction is largely decoupled from the true correction needed. We interpret this as consistent with the model settling into a shallow, low-cost solution in which any small perturbation reduces the training loss without requiring the model to learn content-adaptive structure—a failure mode that is masked when evaluated using only magnitude-based statistics. By enlarging the target residual through intentional degradation, the training objective can no longer be satisfied by such a direction-agnostic solution, which is consistent with the substantially higher sign_match and cosine_sim observed under ID across all three domains.

Across all three domains, sign_match and cosine_sim differences between With ID and Without ID were statistically significant (domain-level paired *t*-tests: *p* < 0.006 for sign_match, *p* < 0.002 for cosine_sim, in every domain). Held-out validation Δ-SSIM reached significance in the Wound domain (*p* = 0.012) but did not reach the 0.05 threshold in ISIC or DRIVE individually (*p* = 0.098, *p* = 0.076). When pooling all three domains (n = 9 seed-domain pairs), all three metrics reached strong significance under both paired *t*-test and Wilcoxon signed-rank test (*p* < 0.005 for all comparisons; Table 5).

**Figure 5:**
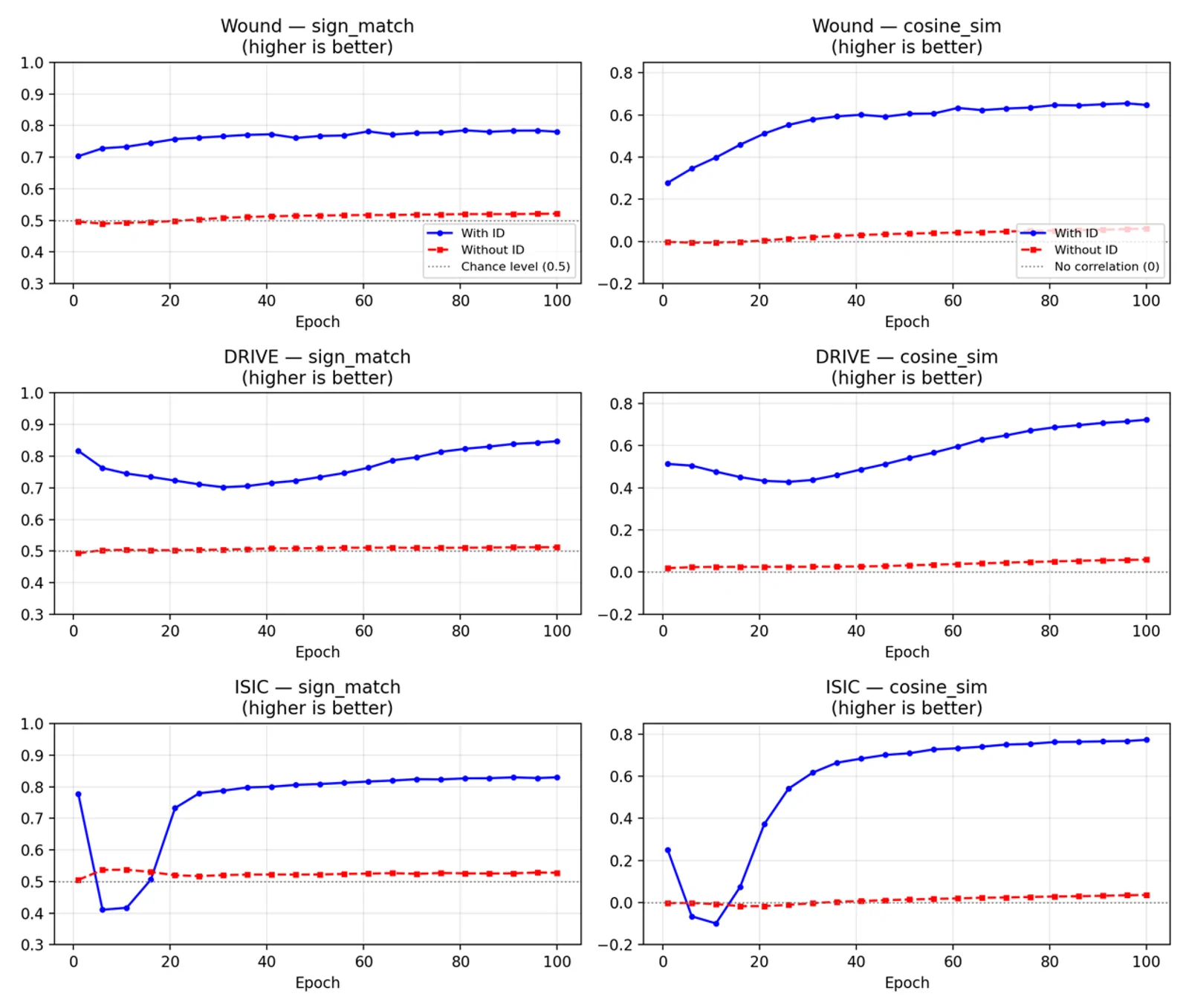
Spatial alignment of predicted residuals across three domains, With vs. Without Intentional Degradation (representative single-seed run, 100 epochs). For each domain, sign_match (**left**) and cosine_sim (**right**) are computed on held-out data at 5-epoch intervals. Without ID (red dashed) converges toward chance-level values (sign_match ≈ 0.5, cosine_sim ≈ 0) in all three domains, indicating direction-agnostic residual prediction; With ID (blue solid) shows sustained improvement toward genuine structural correspondence. See Table 3 for 3-seed mean ± SD at the final epoch.

Across all three domains, Var(Δ)_norm moves closer to 1.0 under intentional degradation relative to Without ID, consistent with more complete recovery of the target residual’s variance (Table 2). The Without ID baseline exhibits high SSIM values in the Wound domain that superficially suggest superior reconstruction quality; however, as shown in Figure 5 and quantified by Δ-SSIM and the spatial alignment metrics (Section 3.3), this apparent quality substantially reflects re-synthesis rather than genuine residual refinement, since the predicted residual’s direction under Without ID is statistically indistinguishable from random (sign_match ≈ 0.5) despite non-trivial residual magnitude.

Δ-SSIM improvements under With ID were observed across all three domains (Wound: 0.491 vs. 0.444; ISIC: 0.464 vs. 0.406; DRIVE: 0.286 vs. 0.097), though the magnitude of the gap varied by domain, with the largest relative gap observed in DRIVE. Because Δ-SSIM alone cannot distinguish a directionally random but non-trivial residual from a genuinely aligned one, the spatial alignment analysis (Section 3.3) provides more direct mechanistic evidence: across all three domains, sign_match and cosine_sim under Without ID converge toward chance-level values, whereas With ID shows consistent, substantial elevation in both metrics.

### 3.4. Robustness and Generalizability

The consistent improvement in Var(Δ)_norm and spatial alignment observed across three fundamentally different medical imaging modalities—wound photography, retinal fundus imaging, and dermoscopy—provides empirical evidence of cross-domain consistency in the evaluated datasets. Notably, the Edge gap varies substantially across domains (2630 for ISIC to 11,794 for DRIVE), yet the method remains effective in all cases, suggesting robustness to varying degrees of distribution mismatch.

While a dedicated, purpose-tuned comparison against existing stabilization strategies remains beyond the scope of this revision, we note that our baseline (Without ID) condition already incorporates a substantial perceptual loss term [7]. A component-wise diagnostic of the training loss revealed that this term dominates the total gradient magnitude by several orders of magnitude throughout training (Section 3.2 and Section 3.3). As an additional check, we reduced this term’s relative weight by approximately four orders of magnitude in a single-domain, single-seed pilot run (Wound domain, 30 epochs), restoring the residual-based loss terms as the dominant gradient contributor; the same direction-agnostic collapse under Without ID and structural alignment under With ID were observed (final sign_match: 0.507 vs. 0.791; final cosine_sim: 0.028 vs. 0.676), indicating that the collapse behavior is not an artifact of a particular loss-weighting configuration. This provides indirect evidence against explaining our results as insufficient perceptual regularization, though we acknowledge this differs from a head-to-head comparison against a dedicated, independently tuned stabilization baseline, which we plan to include in a follow-up revision.

Because intentional degradation enforces model-agnostic distribution alignment, training becomes more stable, delta distribution variance decreases consistently, and the method is immediately applicable to a wide range of coarse-to-fine generative systems without architectural modification.

## 4. Discussion

### 4.1. Why Degradation Works

The key insight is that generators collapse not because they fail, but because the training setup unknowingly rewards them for ignoring the coarse signal. When the target is drastically sharper, richer, and more saturated than the input, an adversarial discriminator assigns large penalties unless the generator re-synthesizes new textures. This creates a destructive feedback loop.

Intentional degradation removes the incentive to ignore the coarse input: the degraded image is now of comparable difficulty, the residual is small and structured, learning focuses on meaningful texture restoration, and the LR input consistently contributes to the output in a stable way.

We hypothesize that the instability of coarse-to-fine refinement pipelines stems from the unbounded distributional gap between the coarse prediction space and the high-resolution target space. Prior work has demonstrated that training instability in generative models is closely linked to the distance between input and target distributions [8,9]. When this gap is unconstrained, the gradient signal becomes directionally ambiguous, leading to the collapse behavior observed empirically. Intentional Degradation addresses this by explicitly compressing the target distribution toward the coarse prediction space—a strategy conceptually related to, yet directionally inverse to, curriculum learning [10,11], which adjusts input complexity rather than target distribution. A formal theoretical characterization of this mechanism, including the relationship between distributional distance and collapse probability, remains an important direction for future work.

As a preliminary illustration of why residual-space metrics were necessary for this study, we highlight a representative dissociation observed in the DRIVE domain: under the Without-ID condition, individual training runs achieved conventional SSIM values in the range of 0.69–0.78 by the final epoch—scores that, taken alone, would suggest reasonably faithful reconstruction—while the corresponding Δ-SSIM remained near zero (as low as 0.02 in one run), indicating that the predicted residual carried little meaningful structural correspondence to the true missing information. This dissociation between a conventional image-level metric and a residual-space metric supports our central methodological claim: SSIM computed on the final reconstructed image can be elevated even when the underlying refinement process has effectively collapsed to near-identity mapping of the coarse input. A full correlation analysis between residual-space and conventional metrics across a larger sample, or a formal expert visual assessment, was not conducted in this study and is identified as a direction for future work.

### 4.2. Relation to Prior Work

Previous solutions to generator instability include feature-wise normalization between stages [12], perceptual losses to constrain generator hallucination [7], and multi-scale discriminators [13,14]. Most of these approaches address collapse-like instability through architectural modification rather than by altering the training target itself, and our method is orthogonal and compatible with all of the above.

The principle of deliberately perturbing the target to prevent a model from collapsing onto a trivial solution has, however, been explored in several adjacent contexts. In diffusion modeling, Cold Diffusion demonstrated that the forward corruption process need not be Gaussian noise and can instead be an arbitrary deterministic degradation (e.g., blur, masking) [15], while Soft Diffusion further developed principled loss formulations and corruption scheduling for general corruption processes [16]. More closely related to the collapse phenomenon addressed here, recent work in sonar image despeckling introduced a statistically constrained loss that explicitly penalizes residual variance collapse in a physics-informed coarse-to-fine refinement pipeline [17]. Our approach shares the underlying goal of preventing a refinement network from settling into a trivial, low-cost solution, but differs in mechanism: rather than imposing an explicit statistical constraint via the loss function, or operating within a multi-step diffusion framework, intentional degradation directly reshapes the training target using empirically profiled domain statistics, within a single-pass GAN-based refinement architecture. We note that most prior work addressing coarse-to-fine collapse outside the diffusion literature has relied on architectural modifications—e.g., attention mechanisms in image inpainting or gating mechanisms in semantic segmentation—rather than target-side interventions, positioning our approach as a relatively underexplored direction within this broader problem space.

Coarse-to-fine refinement pipelines continue to be actively developed within medical imaging specifically; for example, recent work on multimodal retinal image registration adopts an explicit two-stage coarse-then-fine strategy, in which an initial keypoint-based affine alignment is followed by a learned deformable network that further refines the registration locally, with the fine stage stabilized through a combination of content, smoothness, and second-order Laplacian penalty losses [18]. This illustrates that the underlying two-stage coarse-then-fine paradigm remains an active design pattern in current medical image analysis, though this and similar works stabilize the fine stage through loss-level regularization applied to a fixed training target, rather than by reshaping the target distribution itself as in the present work.

### 4.3. Practical Advantages

- No architectural modification required;
- Negligible computation overhead;
- Evaluated within a ConvLSTM-based residual refinement framework; extension to other conditional GAN or diffusion-based refiners remains to be validated;
- Dramatically reduces training instability;
- Empirically grounded parameterization derived from statistical profiling of a single originating domain, shown to remain effective when reused without modification across substantially different domains (Section 3.3).

Beyond training stability, intentional degradation has a direct consequence for clinical realism. Because the generator is trained to predict a bounded residual added onto a plausible coarse image, rather than to synthesize pixel values unconstrained by any input, the range of outputs it can produce is anchored to structures already present in I_deg. This constrains the refined output Ŷ = I_deg + Δ_pred toward modifications consistent with the coarse input’s anatomical layout, reducing the risk of the generator introducing structurally implausible content (e.g., hallucinated wound margins or vessel patterns absent from the coarse prediction)—a known failure mode of unconstrained image-to-image refinement in clinical applications. This structural property is particularly consequential in the DRIVE and ISIC domains, where refined outputs feed directly into diabetic retinopathy and melanoma-screening workflows. We note this as a structural property of the residual-learning formulation rather than a demonstrated outcome: we did not directly verify clinical validity of the refined outputs in this study, and whether this constraint translates into clinically meaningful improvements remains to be established through dedicated evaluation in future work.

### 4.4. Limitations

Several limitations should be acknowledged. First, the wound-healing dataset served both as the originating clinical domain and as the in-domain controlled evaluation setting. The controlled surrogate design—in which LR inputs are derived from the same HR images used to define the ground-truth residual—means that the distributional gap between LR and HR is mathematically determined by D, rather than reflecting the full stochasticity of a real coarse predictor. Although two external modalities (DRIVE and ISIC 2018) were included to evaluate cross-domain transferability, this controlled design means that the surrogate LR distribution is, by construction, only an approximation of the genuine Creator output distribution it is intended to represent. To assess this gap directly, we additionally compared the degraded surrogate (D(HR)) against genuine, uncontrolled Creator outputs on held-out cases (n = 3) using no-reference statistics, since ground truth is unavailable for genuine forecasted outputs. This comparison revealed a non-trivial distributional mismatch: relative to the surrogate distribution used during training, genuine Creator outputs showed a substantially higher mean red-channel value (0.744 vs. 0.624) and a lower mean blue-channel value (0.405 vs. 0.521), indicating that the surrogate degradation does not fully capture the photometric characteristics of real Creator failures.

Despite this gap, the qualitative behavior distinguishing With and Without ID was partially preserved under genuine inputs: the Without ID model produced near-zero corrections to genuine Creator outputs (median correction magnitude approximately one-sixth that of With ID, and zero measured saturation adjustment in all three cases), mirroring the direction-agnostic, low-magnitude failure mode identified in the spatial alignment analysis (Section 3.3) on synthetic held-out data. However, channel-bias values—a proxy for photometric consistency—did not consistently favor With ID (2 of 3 cases), which we interpret cautiously: because the Without ID model applies almost no correction in this regime, its favorable channel-bias values likely reflect an absence of correction rather than an accurate one, making channel bias an uninformative comparison metric in this specific setting. The larger corrections applied by With ID, while structurally more substantive, are not yet demonstrably well-calibrated for the degree of domain shift observed between surrogate and genuine Creator outputs. This finding should be treated as preliminary given the small sample size (n = 3) and the absence of ground truth for genuine forecasted outputs; whether the quantitative gains in Δ-SSIM and Var(Δ)_norm generalize to real coarse predictors at scale remains to be verified in future work with larger genuine-output validation sets.

Second, excessive degradation may under-fit rare textures or clinically subtle high-frequency patterns. Because intentional degradation deliberately compresses the HR target distribution toward the coarse prediction space, overly aggressive degradation may remove information that is difficult for the generator to recover.

Third, the DRIVE dataset (N = 40) is insufficient for large-scale generalization claims and was included solely to verify whether the collapse prevention signal transfers across imaging modalities. Conclusions drawn from the DRIVE results should be interpreted accordingly.

Relatedly, although held-out validation was performed for the refinement generator in this study (Table 3), using image-level splits that were group-aware for the Wound domain to prevent augmentation-induced leakage, the held-out sets remain modest in size (particularly for DRIVE, N = 40), and a single fixed split was used per seed rather than cross-validation. Reporting cross-validated held-out metrics across multiple splits per seed would further strengthen confidence that the observed improvements generalize beyond the specific partitions evaluated here. We further note that n = 3 seeds per domain limits statistical power for per-domain comparisons of magnitude-based metrics (Δ-SSIM); we address this by additionally reporting a pooled analysis across all three domains (n = 9), which supports the same conclusions with substantially higher power.

Fourth, the proposed method requires statistical profiling of the HR–coarse distribution gap prior to degradation parameter selection. Although this constitutes a relatively minor preprocessing step, the effectiveness of intentional degradation depends on how accurately the degradation profile approximates the relevant coarse prediction distribution.

Fifth, generator performance may depend on how well the degradation profile matches the evolution dynamics of the Creator during training. If the Creator output distribution changes substantially across epochs or differs between training and inference, a fixed degradation profile may become suboptimal.

## 5. Conclusions

We introduced Intentional Degradation, a principled method that intentionally degrades high-resolution target images to match the coarse generator’s distribution in two-stage refinement pipelines. This alignment forces the generator to act as a true residual enhancer, preventing re-synthesis behavior and improving structural fidelity, clinical realism, and training stability.

Across three medical imaging domains with distinct modalities—wound-healing photography, retinal fundus imaging, and dermoscopy—the approach consistently reduces delta-map variance, mitigates mode collapse, and maintains stable training when applied to a single ConvLSTM-based residual refinement framework, without requiring modification to this framework’s architecture; generalization to other conditional generative architectures (e.g., diffusion-based refiners) remains to be validated in future work.

Intentional degradation provides a practical target-side perspective: to stabilize refinement models, do not always sharpen the coarse predictions—in conditional coarse-to-fine refinement, sometimes stabilizing the generator requires temporarily reducing target fidelity to match the coarse input distribution. In the clinical wound-healing pipeline that motivated this work, this stabilization directly improves the reliability of the predicted healing trajectory presented to clinicians, reducing the risk that an unconditioned or hallucinated refinement is mistaken for a genuine forecast of wound progression.

## Figures and Tables

**Table 1:** Statistical profiling of HR–coarse or HR–surrogate distribution gaps across three domains.

Domain	HR Edge Energy	LR Edge Energy	Edge Gap	HR Contrast	LR Contrast	HR Saturation	LR Saturation
Wound	6944	1983	4961	0.386	0.278	0.349	0.206
DRIVE	15,419	3625	11,794	0.888	0.536	0.528	0.464
ISIC	4344	1713	2630	0.295	0.197	0.336	0.213

**Table 4:** Epoch-wise evolution of residual-space metrics under intentional degradation, representative single-seed run (Wound domain).

Epoch	Δ-SSIM	Var(Δ)_Norm	Sign_Match	Cosine Similarity	Interpretation
1	0.149	0.720	0.703	0.278	Initial epoch; residual direction already exceeds chance level (0.5)
6	0.137	0.476	0.728	0.346	Directional alignment strengthening despite fluctuating Δ-SSIM
11	0.178	0.523	0.733	0.398	Continued increase in structural correspondence
16	0.194	0.500	0.745	0.459	Alignment consolidating
21	0.251	0.500	0.757	0.512	Clear separation from chance-level baseline established

While early-epoch Δ-SSIM and Var(Δ)_norm fluctuate due to stochastic minibatch effects, sign_match and cosine_sim increase monotonically over this window, indicating that directional (spatial) alignment between predicted and true residuals stabilizes earlier and more consistently than magnitude-based metrics alone. See Section 3.3 for the full-training-run analysis of this effect across all three domains.

**Table 5:** Statistical significance of With ID vs. Without ID differences, by domain and pooled across domains (paired *t*-test on seed-matched pairs, n = 3 per domain, n = 9 pooled).

Domain	Metric	t	*p* (Paired *t*-Test)
Wound	sign_match	41.4	0.0006
Wound	cosine_sim	44.5	0.0005
Wound	val Δ-SSIM	9.0	0.012
ISIC	sign_match	23.5	0.0018
ISIC	cosine_sim	35.7	0.0008
ISIC	val Δ-SSIM	3.0	0.098
DRIVE	sign_match	13.5	0.0055
DRIVE	cosine_sim	29.0	0.0012
DRIVE	val Δ-SSIM	3.4	0.076
Pooled (n = 9)	sign_match	17.6	<0.00001
Pooled (n = 9)	cosine_sim	32.9	<0.00001
Pooled (n = 9)	val Δ-SSIM	4.2	0.0030

Wilcoxon signed-rank test (non-parametric, distribution-free) on the pooled n = 9 sample gave *p* = 0.0039 for all three metrics, consistent with the paired *t*-test results above.

## Data Availability

The DRIVE dataset is publicly available at https://drive.grand-challenge.org/, accessed on 4 April 2026. The ISIC 2018 dermoscopy dataset is publicly available at https://challenge.isic-archive.com/data/#2018, accessed on 4 April 2026. The wound-healing image dataset analyzed in this study is not publicly available due to institutional privacy restrictions but may be made available by the corresponding author upon reasonable request.

## References

[1] Wang X., Yu K., Wu S., Gu J., Liu Y., Dong C., Qiao Y., Loy C.C. ESRGAN: Enhanced super-resolution generative adversarial networks. Proceedings of the European Conference on Computer Vision Workshops (ECCVW).

[2] Burt P.J., Adelson E.H. (1987). The Laplacian pyramid as a compact image code. Readings in Computer Vision.

[3] Ledig C., Theis L., Huszár F., Caballero J., Cunningham A., Acosta A., Aitken A.P., Tejani A., Totz J., Wang Z. Photo-realistic single image super-resolution using a generative adversarial network. Proceedings of the IEEE Conference on Computer Vision and Pattern Recognition (CVPR).

[4] Wang X., Xie L., Dong C., Shan Y. Real-ESRGAN: Training real-world blind super-resolution with pure synthetic data. Proceedings of the IEEE/CVF International Conference on Computer Vision Workshops (ICCVW).

[5] Miyato T., Kataoka T., Koyama M., Yoshida Y. (2018). Spectral normalization for generative adversarial networks. arXiv.

[6] He K., Zhang X., Ren S., Sun J. Deep residual learning for image recognition. Proceedings of the IEEE Conference on Computer Vision and Pattern Recognition (CVPR).

[7] Johnson J., Alahi A., Fei-Fei L. Perceptual losses for real-time style transfer and super-resolution. Proceedings of the European Conference on Computer Vision (ECCV).

[8] Arjovsky M., Chintala S., Bottou L. Wasserstein generative adversarial networks. Proceedings of the 34th International Conference on Machine Learning (ICML).

[9] Gulrajani I., Ahmed F., Arjovsky M., Dumoulin V., Courville A. Improved training of Wasserstein GANs. Proceedings of the Advances in Neural Information Processing Systems (NeurIPS).

[10] Bengio Y., Louradour J., Collobert R., Weston J. Curriculum learning. Proceedings of the 26th Annual International Conference on Machine Learning (ICML).

[11] Karras T., Aila T., Laine S., Lehtinen J. (2017). Progressive growing of GANs for improved quality, stability, and variation. arXiv.

[12] Park T., Liu M.Y., Wang T.C., Zhu J.Y. Semantic image synthesis with spatially-adaptive normalization. Proceedings of the IEEE/CVF Conference on Computer Vision and Pattern Recognition (CVPR).

[13] Wang T.C., Liu M.Y., Zhu J.Y., Tao A., Kautz J., Catanzaro B. High-resolution image synthesis and semantic manipulation with conditional GANs. Proceedings of the IEEE/CVF Conference on Computer Vision and Pattern Recognition (CVPR).

[14] Nah S., Kim T.H., Lee K.M. Deep multi-scale convolutional neural network for dynamic scene deblurring. Proceedings of the IEEE Conference on Computer Vision and Pattern Recognition (CVPR).

[15] Bansal A., Borgnia E., Chu H.M., Li J.S., Kazemi H., Huang F., Goldblum M., Geiping J., Goldstein T. Cold diffusion: Inverting arbitrary image transforms without noise. Proceedings of the Advances in Neural Information Processing Systems (NeurIPS).

[16] Daras G., Delbracio M., Talebi H., Dimakis A.G., Milanfar P. (2023). Soft diffusion: Score matching for general corruptions. Trans. Mach. Learn. Res..

[17] Pillai S., Savner S.S., Sahoo S.K. (2026). Physics-guided self-supervised statistical residual learning for sonar despeckling with improved generalization. IEEE Signal Process. Lett..

[18] Xu J., Zhang S., Shen J., Wei J., Tian S., Yao J., Yan Z., Zhou F. (2025). A coarse-to-fine registration method for multimodal retinal images. Sci. Rep..

